# Impairment of Cognitive Function in Different Domains Early After Lung Transplantation

**DOI:** 10.1007/s10880-021-09787-z

**Published:** 2021-05-19

**Authors:** Roland Tomasi, Mathias Klemm, Christian Ludwig Hinske, Nikolai Hulde, René Schramm, Bernhard Zwißler, Vera von Dossow

**Affiliations:** 1 Department of Anaesthesiology, University Hospital, LMU Munich, Munich, Germany; 2 Clinic of Cardiology, University of Munich, LMU Munich, Munich, Germany; 3 Clinic for Thoracic and Cardiovascular Surgery, Herz- Und Diabeteszentrum NRW, Bad Oeynhausen, Ruhr-University, Bochum, Germany; 4 Institute of Anaesthesiology and Pain Therapy, Herz- und Diabeteszentrum NRW, Ruhr-University Bochum, Bochum, Germany

**Keywords:** Lung transplantation, Cognitive impairment, Postoperative cognitive dysfunction, Delirium, Risk factors

## Abstract

In this prospective observational pilot study patients with the diagnosis of end-stage lung disease and listed for lung transplantation underwent a cognitive function test battery before and after lung transplantation to investigate postoperative cognitive function in three domains (visual and verbal memory, executive functioning, concentration/speed of processing). Additionally we investigated intraoperative risk factors for postoperative cognitive dysfunction. In total, 24 patients were included in this pilot study. The incidence of postoperative cognitive dysfunction was 58.3%. In the cognitive dysfunction group, the domains executive functioning and concentration/attention were significantly impaired whereas memory was not affected. Patients with cognitive impairment had a significantly longer ICU stay. The strongest independent risk factor for the development of cognitive dysfunction was operation time. No influence of cerebral oxygen desaturations on cognitive dysfunction was found. This might have important implications for early psychological rehabilitation strategies in this high-risk patient collective.

## Introduction

Lung transplantation is a well-established therapy for end-stage lung diseases that improves quality of life and survival rates (Yusen et al., 2016). Several studies revealed conflicting results regarding the impairment of cognitive function after lung transplantation (Cohen et al., 2014; Limbos et al., 2000; Smith et al., 2014). Nearly 60% of patients exhibited cognitive impairment as measured by the Montreal Cognitive Assessment Battery (MoCA) (Smith et al., 2014). Above all, 45% of patients with end stage lung disease (excluding patients with cystic fibrosis as leading cause) already have neurocognitive impairment before transplant based on MoCA scores < 26 (Smith et al., 2014). Particularly, hypoxemia is a major contributor to cognitive impairment as shown in patients suffering from chronic obstructive pulmonary disease and regular use of home oxygen seems to be protective (Thakur et al., 2010).

Perioperative neurocognitive disorders included any form of acute event, such as postoperative delirium within the first 72–96 h after surgery, and cognitive dysfunction mostly diagnosed after day seven post procedure, such as delayed neurocognitive recovery and postoperative neurocognitive dysfunction (POCD) (Evered et al., 2018). Delirium is a form of acute brain failure characterized by altered consciousness with a reduced ability to focus, sustain, or shift attention that develops quickly and tends to fluctuate over the course of the day (Inouye, 2006). POCD however is defined by changes in neuropsychological tests administered before and after anesthesia and surgery including memory, executive functioning, and speed of processing and concentration/attention (Murkin et al., 1995). It has significant clinical implications and influences medical compliance (Abildstrom et al., 2000; Smith et al., 2014; Steinmetz et al., 2009). POCD has an estimated incidence of 30–53% at hospital discharge and 5–13% at 3 months for noncardiac and cardiac surgery (Newman, Grocott, et al., 2001; Newman, Kirchner, et al., 2001; Shoair et al., 2015; Silbert et al., 2006) and may persist for years after transplantation influencing quality of life (Newman, Grocott, et al., 2001; Newman, Kirchner, et al., 2001). More importantly, persistent POCD may increase morbidity and mortality (Smith et al., 2018; Steinmetz et al., 2009). Thus, appropriate monitoring and early recognition of POCD in patients may allow appropriate risk stratification and assessment of the efficacy of potentially preventive measures. Intraoperative risk factors for neurocognitive impairment in non-lung transplant procedures are extensive surgery with prolonged anesthesia, high doses of lidocaine and dexamethasone, cerebral oxygen desaturations and aggressive volume replacement (Ntalouka et al., 2018).

Previous studies revealed a significant relationship between low regional cerebral oxygen saturation (rSO_2_) levels and cognitive function in patients undergoing abdominal operation or coronary aortic bypass graft surgery (Casati et al., 2005; Monk et al., 2002; Yao et al., 2004). Cerebral oxygen desaturations are associated with early postoperative neuropsychological deficits in cardiac surgery (Yao et al., 2004) and prolonged hospital length of stay (Edmonds, 2002). Particularly, the prefrontal cortices and the left hippocampus are sensible areas and vulnerable to hypoxia. They represent executive function and verbal memory (Duvernoy, 2013). Interventions to treat rSO_2_ desaturations are associated with less major organ injury, shorter intensive care unit length of stay (John M. Murkin et al., 2007), as well as reduced postoperative delirium (Schoen et al., 2011) and less impairment of neurocognitive function (Yao et al., 2004). Near-infrared spectroscopy (NIRS) allows continuous and noninvasive monitoring of rSO_2_. In lung transplantation 41% of the transplantation centers use the NIRS-based cerebral oximetry during these interventions (Tomasi et al., 2017). However, to the best of our knowledge there is no available literature regarding the relevance of intraoperative rSO_2_ desaturations on development of POCD in lung transplant recipients.

This study used a cognitive function test battery to investigate postoperative cognitive function in three domains (visual and verbal memory, executive functioning, concentration/speed of processing) in lung transplantation recipients. The secondary aim was to explore predictive intraoperative putative risk factors for the development of neurocognitive decline in the early postoperative period after lung transplantation.

## Methods

After institutional review board (nr 354-13, Ethical committee of the Ludwig-Maximilians-University Munich, Germany) approval and written informed consent, 33 patients scheduled for lung transplantation were enrolled in this prospective observational pilot study between March 2014 and September 2016. Baseline demographic data (age, sex, body mass index) and preoperative values describing the severity of the underlying lung disease (lung allocation score, vital capacity, tiffeneau-pinelli index, borg-scale, cardiac output, pulmonary vascular resistance, mixed oxygen saturation and white blood cell count) were collected from the patients record. Intraoperative data was collected from the anaesthesia recording systems. Based on reported risk factors for cognitive dysfunction in non-lung transplant procedures we took the following intraoperative data into account: operation time, intraoperative blood loss, fluid and blood transfusion requirements, and regional cerebral oxygen saturation. In addition, operation specific values, such as type of transplantation, graft ischemia time which causes ischemia reperfusion injury, pulmonary artery clamping and one lung ventilation time as a possible causes of intraoperative hypoxia, the need for catecholamines to ensure adequate perfusion pressure, the use of tranexamic acid and benzodiazepines, the need of additional anesthesia due to re-thoracotomy, and the extracorporeal membrane oxygenation time that causes an inflammatory state were analyzed as possible intraoperative risk factors for cognitive decline. To rule out an acute postoperative neurocognitive disorder, we screened the patients for ICU delirium using Confusion-assessment method (CAM-ICU). Associations of cognitive decline with postoperative variables such as IL 6 as an inflammation parameter, ICU stay and hospital stay were analyzed.

Anesthesia procedures were performed according to the standard operating procedure of the Munich Lung Transplant Group: Patients did not receive oral premedication. General anesthesia was induced with midazolam 0.05 mg/kg, sufentanil 0.5–1 µg/kg, propofol 1 mg/kg and rocuronium 0.5–1 mg/kg and maintained by continuous administration of 5–8 mg/kg/h propofol and 0.7–1.2 µg/kg/h sufentanil. Neuromuscular blockade was achieved by intermittent boluses of 50 mg of rocuronium. All patients were intubated with a left-sided Robertshaw double lumen tube and underwent pressure-controlled ventilation with tidal volumes of 6–8 ml/kg. Analgesia and sedation on ICU were maintained by continuous administration of < 5 mg/kg/h propofol and < 0.5 µg/kg/h sufentanil. We included patients with single and double lung transplantation. As part of the transplant suitability process patients were evaluated by a psychiatrist, evaluating patient and family psychiatric history, addictive substances history, social history and psychiatric diagnosis according to the ICD-10. Patients with preexisting dementia were not eligible to receive a transplant and patients with other psychiatric diagnosis were not eligible for this study. In addition, patients were excluded from participation in the study if they were younger than 18 years of age, if they were on preoperative ECMO, had insufficient knowledge of the German language and hearing or visual impairment.

In the operating room, 2 NIRS sensors (Casmed Fore-Sight sensors, CAS Medical System, Inc., Branford, USA) were applied to the patient’s forehead before the induction of anesthesia. The patient’s baseline rSO_2_ data were acquired before induction while the patient breathed room air or oxygen, depending on the severity of the respiratory failure. Both the right and the left frontal rSO_2_ values were recorded simultaneously. Right- and left-side baseline rSO_2_ values were highly correlated (*r* = 0.770, *p* < 0.001). Therefore, and for a more clinical practicability, the lower value of either side was collected for further analysis. The rSO_2_ data was recorded on the device (Casmed Fore-Sight Elite, CAS Medical System, Inc., Branford, USA). The attending physicians were blinded for the rSO_2_ and therefore no interventions were attempted based on the device values.

Cognitive function test battery to evaluate POCD was performed earliest at day eight after surgery. This is in accordance with the nomenclature recommendations published by Evered et al., 2018 and the ISPOCD study. We set no limit for postoperative POCD evaluation. All patients could be screened before day 30. To guarantee for this unplannable operation that all included patients had the preoperative testing at the same time point before transplantation we performed the preoperative cognitive assessment on the ward before transplantation as part of the patient’s preparation for surgery. The following neuropsychological tests were applied: Visual Verbal learning test (VVL) based on the Rey’s Auditory Recall of words. The VLT assesses the modalities of memory: immediate, consolidated and long-term (Brand & Jolles, 1985). The Stroop Color Word Interference Test (SCWT) (Bohnen et al., 1992), is based on the observation that individuals can read words much faster than they can identify and name colors. The cognitive dimension tapped by the Stroop test is associated with cognitive flexibility, resistance to interference from outside stimuli. It measures cognitive processing and provides valuable diagnostic information on executive functioning. The Concept Shifting Test (CST) can be used to investigate higher cognitive processes including attention, visual recognition, long-term memory, and visual scanning (Van der Elst et al., 2006). We selected the six most appropriate tests for our study: We noted the number of correctly recalled words of the first three rounds and of the fourth round of the VVL and we measured time taken and counted the number of errors of the third round of the CST and of the SCWT. All cognitive function tests were conducted by the same trained research staff member who was never involved in the intraoperative management of the patients.

We used the 20–20 rule to define cognitive decline, (Lewis et al., 2006). The 20–20 rule was applied by comparing a participant’s individual data over the different time points. A patient was classified as having declined on a task if his postoperative score declined in comparison to the baseline score by more than 20%. POCD was detected if performance decreased by 20% on 20% of the tasks. This process is known to not consider the practice effect but was chosen because comparing patients suitable for lung transplantation with a long history of impaired oxygenation with healthy patients as is required for the Reliable Change Index is from our point of view not reasonable. In addition, we screened all patients for postoperative delirium using the Nursing Delirium Scale (Nudesc) (Gaudreau et al., 2005) and the Confusion Assessment Method for the ICU (CAM-ICU) (Ely et al., 2001).

Recorded data was analyzed using Excel (Microsoft, Seattle, WA) to calculate the duration of rSO_2_ below the threshold and the area of the rSO_2_ tracing under the threshold. The rSO_2_ data was analyzed at multiple thresholds (< 60%, < 50% and < 75% from the baseline). Both mean duration below the threshold value of rSO_2_ and the area under the threshold value of rSO_2_ were measured. Patients who had values < 50% were included in the < 60% group as well.

Statistical analyses were performed using IBM SPSS Statistic 24 (SPSS Inc, Chicago, IL) and R 3.4.0 (R Foundation for Statistical Computing, 2016). Statistical comparisons were performed between patients with neurocognitive decline (POCD group) and without decline (non-POCD group). Categorical values are presented as a number (percentage) and were analyzed using Fisher’s two-sided exact test. The Mann–Whitney U test was used to determine differences in the medians of continuous variables, as indicated. P-values were corrected to guarantee a false discovery rate of less than 5% using the Benjamini Hochberg algorithm (Victor et al., 2010). According to the q-values, the following variables were introduced in the multivariate logistic regression analysis to identify intraoperative risk factors for neurocognitive decline: operation time and rate of re-thoracotomy were included. All reported p and q-values are 2-tailed. P and q-values < 0.05 were considered statistically significant.

## Results

**Fig. 1:**
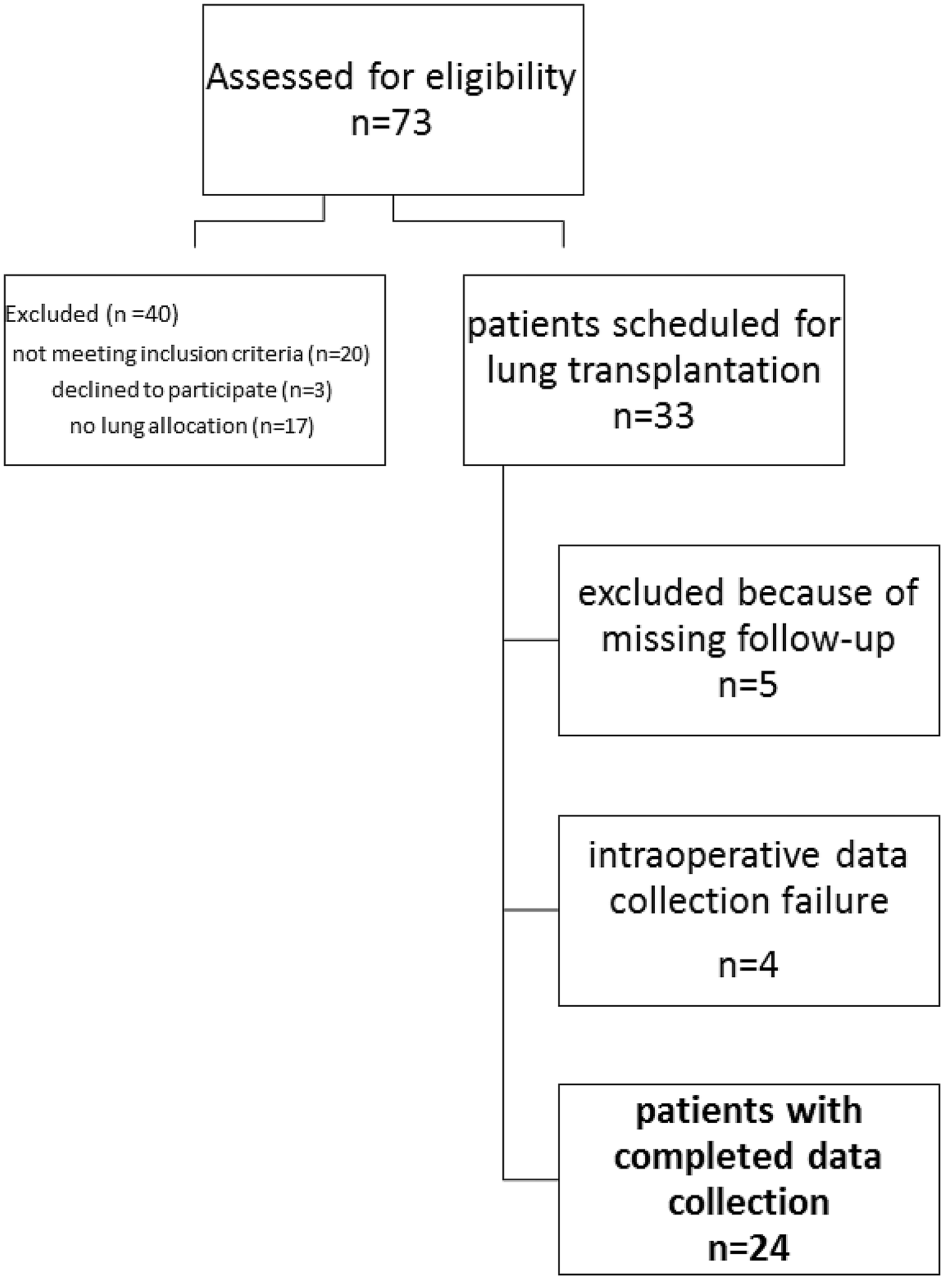
Flow diagram

In total, 73 patients undergoing transplantation were screened according to the inclusion criteria for eligibility. Out of these, 24 were enrolled in the study after written informed consent (Fig. 1). The underlying diagnosis was interstitial lung disease in 20 patients, chronic obstructive pulmonary disease in three patients and cystic fibrosis in one patient. Cognitive dysfunction specific neuropsychological testing was performed preoperatively as part of the patient’s preparation for surgery directly after hospital admission for transplantation and postoperatively not before day 8 and after delirium was ruled out (median at the 16th postoperative day (25th/75th percentile, 8–29 days)). Cognitive decline was detected in our study in 58.3% of patients (*n* = 14). There were no differences in demographic and preoperative characteristics between POCD-Group and Non-POCD-Group patients (Table 1). Significantly more patients with bilateral lung transplantation developed cognitive decline after lung transplantation (*p* = 0.02). All intraoperative and postoperative data of the POCD-Group and NON-POCD-Group are listed in Table 2. Univariate analysis revealed important intraoperative risk factors: Patients with cognitive decline had longer operation time (*q* = 0.044) and required re-thoracotomy more often (*q* = 0.044). In the multivariate analysis, only the operation time (*p* = 0.02) remained as an intraoperative independent risk factor for POCD. Regarding outcome parameters, patients with POCD spent longer time in the ICU (*q* = 0.044) including all readmissions until hospital discharge. Among both groups postoperative delirium was detected in 41.7% with no statistically significant difference between groups. The values of rSO_2_ for POCD are presented in Table 3. The incidence of desaturations < 50% was 41.7%, but there were no significant differences between POCD-Group and NON-POCD-Group patients.

**Table 1:** Demographics and preoperative characteristics of the study groups

	POCD group (*n* = 14)	Non-POCD group (*n* = 10)	*P*	*Q*
Age (years)	55 (50.5/59.0)	59 (48.5/65)	0.312	0.534
Female sex, n (%)	8 (57%)	8 (80%)	0.388	0.534
Body mass index (kg/m^2^)	23 (21.4/26.7)	21.9 (17.9/25.6)	0.371	0.534
Lung allocation score	45.9 (38.6/52.3)	38.8 (35.5/48.8)	0.285	0.534
Vital capacity (%)	38.5 (28.5/45)	41.2 (31.8/45)	0.977	0.977
Tiffeneau-Pinelli index (%)	89 (78.8/91.3)	82 (71.5/89)	0.312	0.534
Borg-Scale	6 (4.5/7.8)	4 (3/8)	0.601	0.735
Preoperative cardiac output (l/min)	5.9 (5.1/6.7)	5.5 (5.0/6.5)	0.695	0.765
Preoperative pulmonary vascular resistance (dyn*sec/cm^−5^)	3.3 (2.9/4.0)	2.8 (2.2/3.3)	0.131	0.534
Preoperative mixed oxygen saturation (%)	72 (68.5/74)	75 (66.5/75.5)	0.357	0.534
Preoperative leukocytes (G/l)	11.8 (9.1/15.4)	8.3 (7.6/12.1)	0.074	0.534

Values are represented as median (25th/75th percentile) significance *p*- and *q*-values < 0.05

*POCD* postoperative cognitive dysfunction

**Table 2:** Patients’ differences in intraoperative and postoperative data for the POCD analysis

		POCD group (*n* = 14)			Non-POCD group (*n* = 10)			*P*			*Q*	
Intraoperative risk factors												
Double lung transplantation, *n* (%)		14 (100%)			6 (60%)			0.020			0.055	
Operation time (min)		317.5 (296.8/397.8)			254.5 (169.8/310.5)			0.004			0.044	
Graft ischemia time (min)		530 (494.0/599.3)			415.5 (363.0/468.8)			0.013			0.055	
Pulmonary artery clamping time (min)		166 (129.5/224.0)			120.5 (98.8/158.3)			0.010			0.055	
One lung ventilation time (min)		232 (167.5/329.0)			157 (126.0/202.0)			0.077			0.121	
Intraoperative blood loss (ml)		3000 (1250/4050)			1650 (925/2550)			0.096			0.140	
Crystalloid requirements (ml)		3459 (2937/4095)			2971 (2128/3542)			0.122			0.157	
Colloid requirements (ml)		625 (200/1525)			500 (75/813)			0.154			0.178	
Red blood cells (ml)		2400 (525/6750)			900 (0/1800)			0.064			0.113	
Fresh frozen plasma (ml)		3125 (1313/6500)			1125 (0/2438)			0.056			0.112	
Platelet concentrate (ml)		600 (0/1500)			0 (0/150)			0.016			0.055	
Tranexamic acid (mg)		2250 (1472/2500)			1484 (0/2000)			0.026			0.063	
Midazolam (mg)		5,43 (3.97/6.89)			4 (3.05/4.95)			0.136			0.166	
Maximum noradrenalin dose (μg/kg/h)		20.5 (18.1/29)			29.6 (19.2/42.1)			0.285			0.313	
Maximum adrenalin dose (μg/kg/h)		4.5 (0./6.7)			6.2 (3.8/12)			0.341			0.357	
Maximum milrinon dose (μg/kg/h)		16.1 (12.2/28.0)			16.5 (10.7/21.5)			0.886			0.886	
Intraoperative ECMO time (min)		172 (58/205)			0 (0/168.3)			0.067			0.113	
Re-thoracotomy, *n* (%)		8 (57%)			0			0.006			0.044	
Association with postoperative variables												
Postoperative delirium, *n* (%)		8 (57%)			2 (20%)			0.104			0.143	
IL-6 on day 2 (pg/ml)		65 (29.3/106.3)			26.7 (16.7/40.2)			0.036			0.079	
Total ICU time until discharge (days)		35 (11.8/64.3)			10 (6.3/11.5)			0.005			0.044	
Hospital stay (days)		59.5 (49/80.8)			46 (29.3/52.8)			0.019			0.055	

Values are represented as median (25th/75th percentile) significance *p*- and *q*-values < 0.05

*POCD* postoperative cognitive dysfunction

**Table 3:** Regional cerebral oxygen saturation

	POCD (*n* = 14)	Non-POCD (*n* = 10)	*P*
Baseline rSO2 (%)	69.5 (67.0/72.0)	70.5 (66.8/72.3)	0.752
Lowest rSO2 (%)	49.5 (45.3/56.3)	52.5 (39.0/63.8)	0.546
Mean duration of rSO2 (min)			
< 60%	20.6 (2.6/49.7)	4.7 (0.1/103.4)	0.585
< 50%	0 (0/3.1)	0 (0/20.9)	0.752
< 75% from the baseline	1.8 (0/14.3)	0.3 (0/48.6)	0.752
Area under threshold (min %)			
< 60%	65.6 (1.2/218.5)	20.6 (0/699.1)	0.796
< 50%	0 (0/0.6)	0 (0/61.1)	0.585

Values are represented as median (25th/75th percentile) significance *p*-values < 0.05

*POCD* postoperative cognitive dysfunction

Cognitive dysfunction specific neuropsychological testing within three domains VVL, SCWT and CST before and after lung transplantation is summarized in Table 4. There were no differences detected in baseline cognitive function tests between POCD-Group and Non-POCD-Group prior transplantation (Table 4). The CST was significantly impaired in the numbers of errors (*p* = 0.006) in the POCD-Group after lung transplantation. The time needed (*p* = 0.002) and the numbers of errors (*p* = 0.016) of the SCWT testing were significantly higher in the POCD-Group.

**Table 4:** Cognitive function test results: baseline and postoperative

	POCD (*n* = 14)	Non-POCD (*n* = 10)	*P*
Baseline			
VVL 1–3	31 (23.8/36.5)	30.5 (23.0/35.5)	0.886
VVL 4	11 (7.0/12.3)	10 (6.8/11.5)	0.472
SCWT3, time (s)	50.8 (45.5/64.8)	48.8 (44.3/78.7)	0.931
SCWT3, error	1 (0/2)	0 (0/4)	0.472
CST3, time (s)	39.4 (32.3/44.2)	49.9 (31.1/64.4)	0.138
CST3, error	0 (0/1)	0 (0/1.3)	0.886
Postoperative			
VVL 1–3	32.5 (26.3/38.5)	28 (24.8/40.0)	0.841
VVL 4	11 (7.5/13.5)	10.5 (8.5/15.0)	0.648
SCWT3, time (s)	73.3 (64.0/119.5)	52 (45.9/57.7)	0.002
SCWT3, error	2 (1/9)	0 (0/2)	0.016
CST3, time (s)	54 (35.7/71.6)	40.4 (31.5/53.6)	0.154
CST3, error	1 (0/5)	0 (0/0)	0.006

Values are represented as median (25th/75th percentile) significance *p*-values < 0.05

*POCD* postoperative cognitive dysfunction

## Discussion

This observational pilot study found that nearly 60% of lung transplant recipients develop cognitive decline in two domains early after surgery. The two domains most affected were executive functioning (SCWT) and speed of processing/attention (CST). The cognitive domain memory (VLT) remained unaffected in both groups. In addition, the duration of surgery is an independent risk factor for the development of POCD, which is associated with longer ICU stay.

Cognitive dysfunction after surgery has to be distinguished between postoperative delirium, which occurs within the first 7 days after surgery and cognitive decline, which is earliest diagnosed at postoperative day 8 (Evered et al., 2018). In our study the overall incidence of postoperative delirium was 41.7% which is comparable to previous studies (34% reported in existing literature) (Smith et al., 2014). However, the rate of delirium did not differ between POCD positive and POCD negative patients. The combination of the risk factor interstitial lung fibrosis in combination with a higher incidence of comorbidities and higher rate of bilateral lung transplantation might have accounted for the higher incidence of delirium in our study compared to other types of operations (Litaker et al., 2001; Noimark, 2009; Smith et al., 2014). In addition, it is well-known that lung diseases per se are an independent risk factor for postoperative delirium (Girard et al., 2008). Delirium is a syndrome of disturbance of consciousness, with reduced ability to focus, sustain, or shift attention that occurs over a short period of time and fluctuates over the course of the day (Inouye et al., 1990). Patients experiencing delirium have a higher probability of death and higher rate of hospital-acquired complications leading to prolonged ICU and hospital stay (Lin et al., 2004). The current literature indicates that delirium is also a risk factor for the development of POCD within the first postoperative week (Rudolph et al., 2008). In this study 80% of the patients with delirium developed POCD. Even if previous studies suggested an association between delirium and POCD in lung transplantation, we could not validate these results in the POCD analysis (Rudolph et al., 2008). Considering the clinical importance of delirium in these high-risk patients, the ability to predict its occurrence would be of value in terms of improving risk stratification.

In our study the incidence of postoperative cognitive decline was 58.3%, which was in accordance with the results of earlier studies in patients undergoing lung transplantation (Smith et al., 2014). Mild forms of POCD in lung transplantation were reported to occur in 67% and moderate forms in 5% of the patients (Cohen et al., 2014). Additionally, 45% of patients had impaired cognitive function before undergoing lung transplantation (Smith et al., 2014). As part of the preoperative evaluation, routine screening for cognitive impairment with an additional comprehensive plan to improve impairment as in patients with chronic kidney disease (Drew et al., 2019) might help to reduce POCD risk in these patients. The POCD incidence after lung transplantation is similar compared to the incidence after cardiac surgery, where incidences between 30 and 65% were reported (van Harten et al., 2012). Cognitive deterioration after lung transplantation, including a depressed level of consciousness and impairments of attention, memory, and reaction time, is an important issue. Persistent cognitive dysfunction may affect patients compliance at taking immunosuppressive medication (Campbell et al., 2012; Stilley et al., 2010), quality of life and may even be an indicator of adverse outcome in these patients (Abildstrom et al., 2000; Smith et al., 2014; Steinmetz et al., 2009). In our multivariate analysis the operation time was revealed as an independent risk factor for POCD. In the univariate analysis, the additional need of anaesthesia due to the necessity of revision operations for postoperative complications seems to augment the risk of POCD. This result agrees with the finding of the ISPOCD1 study, in which the necessity of multiple operations was a risk factor for POCD (Moller et al., 1998). Experienced surgeons with resulting faster operation times and fewer postoperative complications could significantly reduce the risk of POCD. Longer operation time and the necessity of more than one operation aggravates the inflammatory response (Cohen et al., 2014; Krenk et al., 2010), which is a risk factor in the pathogenesis of POCD (Krenk et al., 2010). In this context, it should be mentioned that IL-6 plasma levels were significantly increased on postoperative day two in the POCD-group. This might be an association between the systemic inflammation and persistent neuroinflammation often discussed in the context of POCD. This is underlined by the fact that before adjusting for p-values, longer graft ischemia time was associated with POCD. It has previously been described to be an independent risk factor for POCD (Cohen et al., 2014). After adjustment of the p-values this factor was no longer significant for POCD. Univariate analysis revealed that POCD is associated with a prolonged total ICU stay including readmissions. This is associated with significant implications as recovery and rehabilitation process after lung transplantation are delayed.

This is the first study that monitored cerebral oximetry in patients undergoing lung transplantation in context with a cognitive test battery pre- and postoperatively evaluating three different domains of cognition. Interestingly, we found no influence of rSO_2_ on POCD development. The baseline rSO_2_ was not different between the groups. The median duration of desaturations < 60% was longer in the POCD group, but this was not statistically significant. Desaturation periods < 50% were even longer in the NON-POCD group. Our results are in contrast to cardiac surgery patients, where patients with intraoperative rSO_2_ desaturations < 50% are reported to have a significantly higher incidence of POCD (de Tournay-Jetté et al., 2011; Slater et al., 2009). In this study, desaturations < 75% from the baseline occurred in 58.3% of the patients and desaturations < 50% in 41.7%. In cardiac surgery, desaturations < 50% are more frequent in patients with POCD when compared to patients without POCD (Slater et al., 2009). A possible explanation might be long-term impaired oxygenation in end-stage lung diseases: these patients seem to be less vulnerable to develop POCD in case of intraoperative desaturations during one-lung ventilation. Additionally, shorter desaturation periods < 60% (45 min%) compared with periods reported in non-transplant surgeries (1322 min%) (Kim et al., 2016) may explain the different results regarding the influence of rSO_2_ desaturations on POCD. Furthermore, our data indicate no deterioration in outcome for patients with desaturations < 50%. We observed solely a prolonged ventilation time in patients with desaturations < 75% from the baseline.

Even if the cognitive domains verbal memory and executive function seem to be most affected in hypoxic states, our data suggests that verbal memory was not affected, neither in POCD-group nor in NON-POCD-group. However, we detected significant alterations in executive functioning and resistance to interference from outside stimuli (SCWT) as well as attention and concentration (CST). Our findings have significant implications for the post-transplantation management. Cognitive impairment results in irregular uptake of immunosuppressive medications (Rodgers et al., 2018; Stilley et al., 2010) which increases the risk of graft loss (Kuypers, 2020). Therefore, a cognitive dysfunction specific neuropsychological testing before and after transplantation could be helpful to assess patient-individualized cognitive resources and to implement early strategies for rehabilitation depending on the impaired cognitive domain.

Our observational pilot study has several limitations: First, the number of observed patients was small, larger randomized controlled studies are necessary to confirm our preliminary data. Secondly, as lung transplantation procedures are often performed at night time, we cannot exclude that the distortion of the circadian rhythm might have affected the performance of neuropsychological tests prior lung transplantation. Thirdly, postoperative cognitive decline might be influenced by immunosuppressive therapy with tacrolimus in all patients (Pflugrad et al., 2018). Besides tacrolimus, postoperative pain management with a pronounced need for intravenous opioid medication due to the surgical trauma may be an additional risk factor for POCD because it may influence level of alertness and concentration (Wang et al., 2007). The use of regional anaesthesia may help to reduce this risk. However, the cumulative intraoperative administration of sufentanil did not differ significantly between patients with and without POCD in our study.

Furthermore, the chosen method for detecting POCD was the 20–20 method. Previous studies confirmed high sensitivity for this method (Lewis et al., 2006), although the reliable change index (RCI) has the best combination with respect to sensitivity and specificity. However, the RCI needs a control group to be calculated. A control group without surgery limits the interpretation of our results because the surgical stimulus is missing. In addition, end-stage lung disease patients not listed for transplantation are not comparable to listed candidates. Every lung transplantation recipient has an individual progress of his disease and different adaptation to hypoxia and hypercapnia.

We conclude that a cognitive function test battery revealed alterations of cognitive function in the domains executive functioning and attention/concentration in patients developing cognitive decline after lung transplantation whereas memory was not affected. This was not associated with intraoperative rSO_2_ desaturations. Multivariate analysis revealed operation time as an independent risk factor. This might have important implications for patient-individualized rehabilitation strategies for different cognitive domains during the transplantation process, such as therapy of chronic hypoxemia, avoidance of sedating medications and polypharmacy, treatment of depression, exercise, brain training (Song et al., 2019), and screening and treatment of delirium.
